# Gender Dysphoria – Prevalence and Co-Morbidities in an Irish Adult Population

**DOI:** 10.3389/fendo.2014.00087

**Published:** 2014-06-13

**Authors:** Ciaran Judge, Claire O’Donovan, Grainne Callaghan, Gadintshware Gaoatswe, Donal O’Shea

**Affiliations:** ^1^Department of Endocrinology, St. Columcille’s Hospital, Dublin, Ireland; ^2^Department of Endocrinology, St. Vincent’s University Hospital, Dublin, Ireland

**Keywords:** gender dysphoria, gender identity disorder, transgender, gender reassignment surgery, gonadectomy, hormone therapy

## Abstract

**Introduction:** Gender dysphoria (GD) is a condition in which there is a marked incongruence between an individual’s psychological perception of his/her sex and their biological phenotype. Gender identity disorder was officially renamed “gender dysphoria” in the DSM-V in 2013. The prevalence and demographics of GD vary according to geographical location and has not been well-documented in Ireland.

**Methods:** We retrospectively reviewed medical records of 218 patients with suspected or confirmed GD referred to our endocrine service for consideration of hormonal therapy (HT) between 2005 and early 2014. We documented their demographics, clinical characteristics, and treatment during the study period.

**Results:** The prevalence of GD in the Irish population was 1:10,154 male-to-female (MTF) and 1:27,668 female-to-male (FTM), similar to reported figures in Western Europe. 159 of the patients were MTF and 59 were FTM, accounting for 72.9% and 27.1% of the cohort, respectively. The rate of referral has increased year-on-year, with 55 patients referred in 2013 versus 6 in 2005. Mean ages were 32.6 years (MTF) and 32.2 years (FTM). 22 of the patients were married and 41 had children, with 2 others having pregnant partners. 37.6% were referred by a psychologist, with the remainder evenly divided between GPs and psychiatric services. There were low rates of coexistent medical illness although psychiatric conditions were more prevalent, depression being a factor in 34.4% of patients. 5.9% of patients did not attend a mental health professional. 74.3% are currently on HT, and 9.17% have had gender reassignment surgery (GRS). Regret following hormonal or surgical treatment was in line with other Western European countries (1.83%).

**Conclusion:** The incidence of diagnosis and referral of GD in Ireland is increasing. This brings with it multiple social, health, and financial implications. Clear and accessible treatment pathways supported by mental health professionals is essential.

## Introduction

Gender dysphoria (GD) is a recognized condition in which there is marked incongruence between an individual’s psychological perception of his/her sex and their biological phenotype (1). GD has replaced the term gender identity disorder (GID) in the 2013 publication of the DSM-V, which itself replaced “transexualism” in 1980. The cause is not well understood and is believed to be due to a complex interaction of psychosocial and biological factors during development (2, 3).

Prevalence varies based on geographical location, with higher rates in Western Europe and America (0.001–0.002%) (4–6) compared to lower rates in Japan (0.0009%) (7). In most studies, male-to-female (MTF) GD cases tend to significantly outnumber female-to-male (FTM) cases (7).

The DSM-V has strict criteria for the diagnosis of GD that differ between children and adults. However, both include a persistent and strong conviction that they are the wrong sex, feel discomfort, distress, or anxiety with their gender-role and sexual characteristics, and possess a strong and persistent desire to be a member of the opposite sex (1). These criteria are based on the assumption that GD is a purely psychiatric condition which is a topic of controversy.

The complex nature of GD requires that psychological, social, and medical issues are addressed by a multidisciplinary team.

The Department of Endocrinology in St. Columcille’s Hospital (SCH), Dublin, has been managing patients with GD since 2000. Patients are referred for consideration of hormonal therapy (HT) by a mental health professional or GP in the majority of cases. We have previously reported the characteristics of the first 52 patients referred for HT in SCH (8), however, GD in Ireland remains poorly investigated. This paper aims to update the characteristics of GD in Ireland, show the current prevalence of the condition, and discuss the need for developing accessible, well-defined pathways of care.

## Materials and Methods

We retrospectively reviewed the medical records of 218 patients referred to the GD clinic between 2005 and early 2014. A diagnosis had been made by a MHP based on DSM-IV/V criteria in most instances, however, some patients required a referral from the clinic to a mental health professional if this had not occurred This initial consultation included a full medical and surgical history and examination as well as baseline laboratory investigations; full blood count, renal, liver, and bone profiles, fasting lipids, and glucose as well as hormonal profiling.

Data collection focused on documentation of past medical and surgical history (including hypertension, coronary artery disease, thromboembolic disease, dyslipidemia, diabetes mellitus, etc.), psychiatric background (including depression and autistic spectrum disorder etc.), and family history (including cardiovascular conditions etc.). Smoking and alcohol use were also recorded. GD-specific questioning aimed to establish age at self-diagnosis, time spent in desired role, and hormonal or surgical therapy prior to presentation, etc (Table 1).

**Table 1:** **Demographics and co-morbidities**.

Variable	MTF	FTM
Number	159	59
Age (years) (SD)	32.5(13.26)	32.2(11.40)
Married (previous or current)	41	5
Number with children	37	3
Prior hormone therapy	36	6
Living in role	66	44
**MEDICAL HISTORY**		
Hypertension	17	3
Dyslipidemia	42	5
Diabetes	1	2
Cigarettes	40	17
**PSYCHIATRIC CO-MORBIDITY**		
Depression	55	19
Schizophrenia	8	−
Bipolar affective disorder	2	2
Deliberate self-harm/suicide attempt	13	6

*SD, standard deviation; MTF, male-to-female; FTM, female-to-male*.

Consideration for HT required that patients were diagnosed by a minimum of one, preferably two mental health professionals.

In our service, we aimed to initiate HT at the second visit, thus this necessitated that a detailed discussion with the patient regarding the risks and benefits of HT be undertaken at the initial consultation. Providing there were no contraindications and the patient received a definitive diagnosis of GD from an appropriate mental health specialist, HT could commence on the second consultation. We also analyzed the incidence of regret or adverse effects to treatment in those presenting for follow-up. Those wishing to proceed with gender-related surgery were referred to appropriate specialists in Ireland or abroad.

## Results

### Demographics

**Figure 1:**
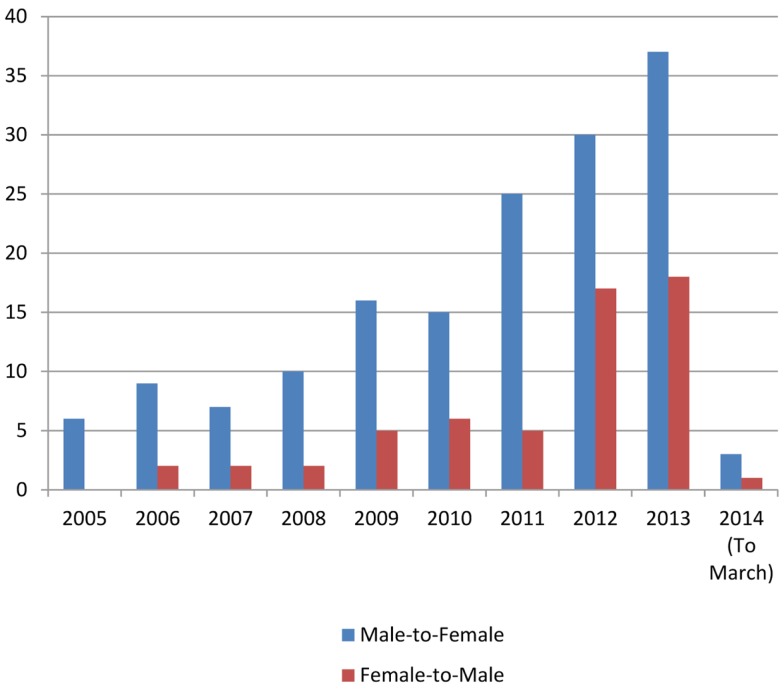
**Number of referrals per year**.

In the study, 218 patients with GD were referred to our clinic between 2005 and early 2014 (Table 1). 72.9% of these were MTF and 27.1% FTM, with a MTF:FTM ratio of 2.7:1. Based on the 2011 census reports (9), this indicates a prevalence of GD in 1:14,756 people (0.0067% of the population), categorized into 1:10,154 MTF (0.0098%) and 1:27,668 FTM (0.0036%). Seventy-one percent of the patients were Irish, with Leinster providing the most patients when subdividing the cohort based on region. The numbers of patients being referred increased steadily over the 9 years study period, from 6 referrals in 2005 to 55 in 2013 (Table 2; Figure 1).

**Table 2:** **Number of referrals per year**.

Year of referral	MTF	FTM	Total
2005	6	−	6
2006	9	2	11
2007	7	2	9
2008	10	2	12
2009	16	5	21
2010	15	6	21
2011	25	5	30
2012	30	17	47
2013	37	18	55
2014 (to March)	3	1	4

*MTF, male-to-female; FTM, female-to-male*.

The average age at referral was similar between both gender groups at 32.6 years for MTF and 32.2 years for FTM, ranging from 15 to 69 years. There was a clear trend in the study showing people are being referred at younger ages as time goes on. The average age at referral from 2005 to 2009 inclusive was 36.77 years, compared with the average age between 2010 and 2013, which was 30.14 years. Twenty-one percent of the patients were married at presentation or in the past, and 18.8% reported having children. Two patients reported having pregnant partners, one of whom conceived using banked sperm.

One hundred thirty-five patients (61.9%) declared their age at self-diagnosis of GD as being pre-pubertal, with 14.7% stating they realized their issues around gender as adolescents. 50.9% of patients were living in their desired gender-role on a full-time basis, with approximately one quarter of patients not living in role prior to initial presentation.

The majority of referrals were made by a psychologist (37.6%), with the remainder evenly divided between psychiatrists and GPs. Although most patients were seen by at least one MHP, 5.96% of patients did not attend either a psychiatrist or psychologist. Of these, five patients received HT without prior MHP consultation, however, one patient obtained the medications via the internet and three others were on HT and also had gender-related surgery prior to presentation. Forty-four patients (20.2%) were on HT prior to initial visit to the GD clinic, 9 of whom ordered it via the internet, 16 individuals sourced hormones from practitioners either in Ireland or the UK, 9 sourced it from overseas, and in the case of the remainder the source was unknown.

### Medical and psychiatric co-morbidities

Several patients had co-existing medical co-morbidities, with dyslipidemia (21.6%), asthma (11.45%), and hypertension (9.17%) being the most prevalent. Twenty-six percent of the patients reported being smokers at the time of presentation, with 23.85% having smoked regularly in the past. Twelve patients consumed more than 15 units of alcohol per week, however, eight patients (3.67%) reported abusing alcohol to a damaging degree in the past. Seventy-five (34.4%) patients had a history of depression, 25.3% of those admitting to acts of deliberate self-harm. Other psychiatric conditions encountered included schizophrenia (3.67%), bipolar disorder (2.29%), and Asperger’s syndrome (2.29%) (Table 1).

Blood samples were taken as they are important to both assess initial risk and evaluate possible future adverse events, including cardiovascular disease (10). Twenty-five percent were found to have an initial fasting cholesterol of >5.3 mmol/L and 31% had a fasting LDL of >2.6 mmol/L, both of these figures being the upper reference range for the laboratory in SCH. Of these, 47 were known to have dyslipidemia (42 MTF versus 5 FTM), 12 people went on to commence lipid-lowering therapy, with others initiating diet control or having a repeat sample within normal ranges. Five people were found to have a fasting blood glucose of >5.9 mmol/L, however, none were found to have diabetes mellitus on repeat testing (Table 1). Nearly 20% of patients had a family history of vascular disease including myocardial infarction (9%), deep venous thrombosis (2%), and cerebrovascular disease (9%).

### Post presentation

To date, 55 patients (22.3%) have undergone surgery, 36 (65.5%) of these have been MTF patients. Mammoplasty was the most common procedure, with 19 (52.8%) of those undergoing surgery electing to have breast augmentation. Twenty (36.4%) surgical patients opted for gender reassignment surgery (GRS), which constitutes 9.17% of the total cohort. A very small number also had facial or laryngeal surgery for voice alteration (Table 3). As there are currently no GRS services in Ireland, patients are referred to a variety of centers in the United Kingdom and elsewhere. Additionally, there are currently 11 patients referred to Charing Cross Hospital for consideration of GRS.

**Table 3:** **Surgical procedures in gender dysphoria patients**.

Variable	MTF	FTM
GRS	15	9
Breast surgery	16	19
Facial surgery	6	−
Laryngeal surgery	2	−

*GRS, gender reassignment surgery*.

Dual energy X-ray absorptiometry (DEXA) scanning is now part of the standard screening protocol of patients presenting to our clinic. In our cohort, 39 patients (17.9%) underwent this investigation at time of last known follow-up, with 20.5% of those studied discovered to have osteopenia, and 7.7% diagnosed with osteoporosis.

Thirty-seven patients (17%) reported adverse effects following hormonal or surgical therapy. Eight patients reported decreased mood or libido, six patients were found to have developed dyslipidemia, five patients had hot flushes/headaches, and one patient developed a fistula post GRS. Four patients (1.8%) expressed regret following treatment. All of the incidences of regret involved MTF, three of who were post GRS with the other in the hormonal phase of treatment.

## Discussion

In this retrospective study, we characterized GD patients attending an endocrine unit in the Republic of Ireland. From this study, the prevalence of GD is 1:10,154 MTF (0.0098%) and 1:27,668 FTM (0.0036%). This is comparable to the prevalence of GD reported in Western Europe, which ranges from 1:11,900 to 1:18,000 for MTF and 1:30,400 to 1:54,000 for FTM (4, 10–12). Our analysis echoed similar international studies in that we collated data from a single specialist GD clinic. De Cuypere et al. reviewed 10 such studies spanning 39 years with prevalence very much matching our own (10). The prevalence rate of GD in our cohort is likely to reflect that of the Republic of Ireland as a nation, as our unit has been the main specialist center in the country over the study period.

In the seventh version of the World Professional Association for Transgender Health (WPATH) Standards of Care for the Health of Transsexual, Transgender, and Gender-Non-conforming People (13), it is highlighted that in more recent analyses there appears to be a higher prevalence of GD, possibly reflecting an increase in the number of people seeking help from healthcare professionals (13–15). This hypothesis is supported by our analysis, which shows 6 referrals to our service in 2005 versus 55 in 2013.

Our MTF/FTM sex ratio of 2.7:1 is similar to the ratio of 3:1 reported in our previous study (8) and the sex ratio reported in Western Europe (10, 11). It is in line international data ranging from 3 to 5:1 (16).

The age of initial assessment was the same for both MTF and FTM (32 years). This is significantly different to our previous figures which indicated FTM patients presented, on average, 8 years younger than MTF (8). Age of presentation for MTF has decreased dramatically (39 versus 32.6) while that of FTM patients has remained approximately the same (31 versus 32.2) (8). The reason for this is unclear but may represent the condition now being more socially acceptable facilitating earlier presentation. This age is in line with a Belgian study, which showed a mean age of initial presentation of ~30 years (10). Over 60% of patients declared their age at self-diagnosis to be pre-pubertal which highlights the need for early recognition and diagnosis of the condition.

21% of the patients were married at presentation or in the past, and 18.8% reported having children. This is lower than the reported rates in similar European studies (10). Two of the patients had pregnant partners, one of whom conceived using banked sperm.

The rate of co-existing medical illness such as hypertension and diabetes were low in GD patients, which could perhaps be expected as the patients are generally young (average age 32 years). It is important to identify and optimize control of these medical co-morbidities prior to initiating the hormonal phase of treatment, as they can be exacerbated by hormones such as estrogen (17–19). Close attention should be paid to the possibility of cardiovascular events, as accruing evidence has suggested that MTF GD patients may be at an increased cardiovascular risk (20–22). Twenty-six percent of patients were smokers at initial presentation which is in line with the national average of 29% (23). Patients were informed that they would not be commenced on HT until they had ceased smoking due to the increased risk of venous thrombosis (24).

Depression was common to 34.4% of patients, while the incidence of other major psychiatric illnesses was relatively low. A survey of Dutch psychiatrists regarding psychiatric co-morbidity in those with GD found that 61% of their cohort had a co-existing psychiatric disorder (25). The presence of such a high rate of co-morbid psychiatric illness underlines the need for a well-defined and rigidly adhered-to mental health consultation service. While our rate of patients who did not attend a MHP (5.96%) is low, it outlines the challenges faced by specialist centers in ensuring that patients with GD receive the appropriate mental health support in the setting of increased referrals.

Currently, there are no centers in Ireland which provide GRS, which may have contributed to only 11% of our patients undergoing sex reassignment surgery. However, it is worth noting that there is an agreement between the Irish Health Service Executive and certain GRS centers in other countries to facilitate ease of access for Irish patients wishing to undergo GRS. Unfortunately, this system is somewhat convoluted and frequently causes delays in access to surgery. This is a frustration experienced by many of the patients examined in our study.

The regret rate following both HT and GRS was low with four (2%) patients expressing regret (all MTF). This is slightly higher than previously reported figures of 1–1.5% for MTF (26, 27). Three of these had gender-related surgery and one had received hormones as the only treatment. Although low, the regret rate of 2% illustrates the need for continued input from mental health professionals beyond GRS. Unfortunately, these services are not always readily available, and this is a matter for further discussion.

## Conclusion

The prevalence of GD in the Republic of Ireland is comparable to that reported in Western Europe. However, the true prevalence may be difficult to assess due to patient worries regarding stigma and societal acceptance of the condition. The number of patients treated by our unit is increasing each year, which will pose considerable challenges with already limited resources. The liaison with mental health services is of critical importance to patients and to the success of treatment. Difficulties in obtaining ongoing mental health care are all too common and lead to the burden of care resting on the endocrinology service. It is vital that a well-defined and accessible treatment pathway is continually developed in order to ensure a gold standard of care for patients with GD.

## Conflict of Interest Statement

The authors declare that the research was conducted in the absence of any commercial or financial relationships that could be construed as a potential conflict of interest.
